# Age-dependent and region-specific alteration of parvalbumin neurons, perineuronal nets and microglia in the mouse prefrontal cortex and hippocampus following obesogenic diet consumption

**DOI:** 10.1038/s41598-021-85092-x

**Published:** 2021-03-10

**Authors:** Amy C. Reichelt, Claire A. Lemieux, Oren Princz-Lebel, Ashmita Singh, Timothy J. Bussey, Lisa M. Saksida

**Affiliations:** 1 Department of Physiology and Pharmacology, Schulich School of Medicine and Dentistry, Western University, London, ON Canada; 2 Robarts Research Institute, Schulich School of Medicine and Dentistry, Western University, London, ON Canada; 3 Brain and Mind Institute, Western University, London, ON Canada; 4 Neuroscience Graduate Program, Schulich School of Medicine and Dentistry, Western University, London, ON Canada; 5 Florey Institute of Neuroscience and Mental Health, Melbourne, VIC Australia; 6 Present Address: Department of Medical Sciences, University of Adelaide, Adelaide, SA 5005 Australia

**Keywords:** Development of the nervous system, Diseases of the nervous system, Feeding behaviour, Learning and memory, Obesity

## Abstract

Emergent evidence demonstrates that excessive consumption of high fat and high sugar (HFHS) diets has negative consequences on hippocampal and prefrontal cortex (PFC) function. Moreover, the delayed maturation of the PFC including the late development of parvalbumin-expressing (PV) interneurons and perineuronal nets (PNNs) may promote vulnerability to HFHS diet-induced nutritional stress. However, the young brain may have some resistance to diet-induced neuroinflammation. Thus, we examined the impact of a HFHS diet commencing either in adolescence or adulthood in male mice. PV interneurons, PNNs and microglia were assessed using immunohistochemistry. We observed greater numbers of PV neurons and PNNs in the hippocampus and the prelimbic and infralimbic PFC in adult mice in comparison to our younger cohort. Mice that consumed HFHS diet as adults had reduced numbers of hippocampal PV neurons and PNNs, which correlated with adiposity. However, we saw no effects of diet on PV and PNNs in the PFC. HFHS diet increased microgliosis in the adult cohort, and morphological changes to microglia were observed in the PFC and hippocampus of the adolescent cohort, with a shift to activated microglia phenotypes. Taken together, these findings demonstrate different regional and age-specific effects of obesogenic diets on PV neurons, PNNs and microglia.

## Introduction

Unhealthy diets with large proportions of foods high in refined sugars and saturated fats are prevalent globally^1^, particularly among adolescents and young adults^2^. Excessive consumption of foods high in saturated fat and refined sugar (HFHS) contribute to pathological weight gain and obesity. Moreover, it is now recognised that obesity and HFHS diets have detrimental impacts on the brain^3^. Due to its delayed development when compared to other areas of the brain, the adolescent prefrontal cortex (PFC) is considered a region particularly sensitive to the negative effects of HFHS diets and obesity^4–7^. Moreover, the hippocampus—a critical brain region for learning and memory—is functionally altered by the excessive consumption of HFHS diets^8^. The maturation of the hippocampus is generally complete by late childhood^9^, although some refinement is likely still continuing into early adolescence^10,11^. Hippocampal-dependent memory in people and rodents is rapidly impaired by consumption of unhealthy diets^12–16^, indicating that the hippocampus may be vulnerable to negative consequences of HFHS-diets, irrespective of age.

The neural mechanisms that initiate HFHS-diet induced cognitive impairment are yet to be fully elucidated. Emergent evidence indicates that specific neuronal subtypes involved in cognition and associated cells may be particularly vulnerable to HFHS diets. For example, fast-spiking GABAergic inhibitory interneurons expressing the calcium-binding protein parvalbumin (PV) are critical for cognitive function and also have delayed maturation trajectories within the PFC^17^. In the cortex, PV neurons regulate excitatory-inhibitory balance and high frequency neuronal synchronisation, and their dysfunction is associated with cognitive impairments including in attention and working memory^18–20^. As PV neurons mature and integrate into neural networks in the cortex, the expression of PV-protein and mRNA increases along with distinct electrophysiological properties, and this is also reflected by their increased immunoreactivity and numbers during the process of brain maturation^21,22^. Consumption of high-energy “obesogenic” diets is associated with reduced cortical and hippocampal GABA concentration^23^ and decreased PV neuron immunoreactivity in regions of the frontal cortex^24–27^ and hippocampus in rats^25,28^, indicating that PV neurons may be at risk of HFHS diet-induced dysfunction. However, due to the relatively late development of PV neurons in the PFC^17,22^, environmental challenges during early life such as stress or poor nutrition may have pronounced and enduring effects on these neurons that could precipitate the development of neuropsychiatric conditions^29^.

Another maturational event is the development of specialized extracellular matrix structures called perineuronal nets (PNNs), which frequently surround cortical PV neurons^30^. These mesh-like structures are composed of chondroitin sulfate proteoglycans with a hyaluronic acid backbone, which are secreted by cell membrane bound hyaluronic acid secretase^31^. Cortical PNNs mature during the juvenile-adolescence period to form a protective microenvironment around neurons and provide synaptic stabilisation that modulates plasticity^32–37^. Envelopment of PV-neurons by PNNs is linked to their maturation, as PNNs restrict plasticity and can stabilise synapses^37^. This is often determined as a key event in the closure of the ‘critical period’ of heightened neuroplasticity in brain development, and pharmacological removal of PNNs has been shown to reinstate the juvenile-like levels of plasticity in the cortex^38^. The prolonged maturation of PNNs has been noted within the frontal cortices^17^ and the hippocampus^39^ , therefore, PV neurons in the adolescent brain may be more vulnerable to obesity-evoked alterations to the extracellular environment as PNNs have not fully formed their protective microenvironments^26,27,40,41^.

Obesity is also associated with chronic inflammation due to the systemic release of inflammatory cytokines by adipocytes^42^, which then enter the brain via the blood brain barrier causing neuroinflammation^43^. The effects of obesogenic diets on neuroinflammation might occur in an age-dependent manner, with younger rats being more resistant to obesity-induced neuroinflammation, indicated by an increase in cortical interleukin-1β levels in 18-month-old, but not 3-month-old rats^44^. What has not yet been examined, however, is the role of microglia in HFHS-induced pathology across age groups. Microglia are critical immune cells in the brain that change in response to the presence of neuroinflammatory cytokines^45,46^, to an active state, reflected by phenotypical morphological alterations^47^. Although microglia are a key defence mechanism within the brain, their activation can result in damage to PNNs through either the release of proteolytic enzymes such as matrix metalloproteases (MMPs)^48^ or by physically interacting with these structures^49^, which may evoke downstream neuronal dysregulation^50,51^.

Changes to PNN structures, PV neurons and microglia following the consumption of HFHS diets are likely tightly associated, but have yet to be studied concurrently in a controlled setting. The goal of this study was to examine the effects of HFHS diets on PV neurons, PNNs and microglia density/morphology in regions of the mouse analogue of the frontal cortex and hippocampus of adolescent or adult male mice. In this way, we were able to provide direct comparisons of effects regarding the impact of obesogenic diets when consumed at different neurodevelopmental stages. This HFHS diet recapitulates an obesogenic “western” diet consumed by many people worldwide and therefore allowed us to determine if PV neurons, PNNs and microglia within the young brain have differential susceptibility to nutritional stress caused by this diet in comparison to the adult brain.

## Results

### HFHS diet induced weight gain and increased adiposity

**Figure 1:**
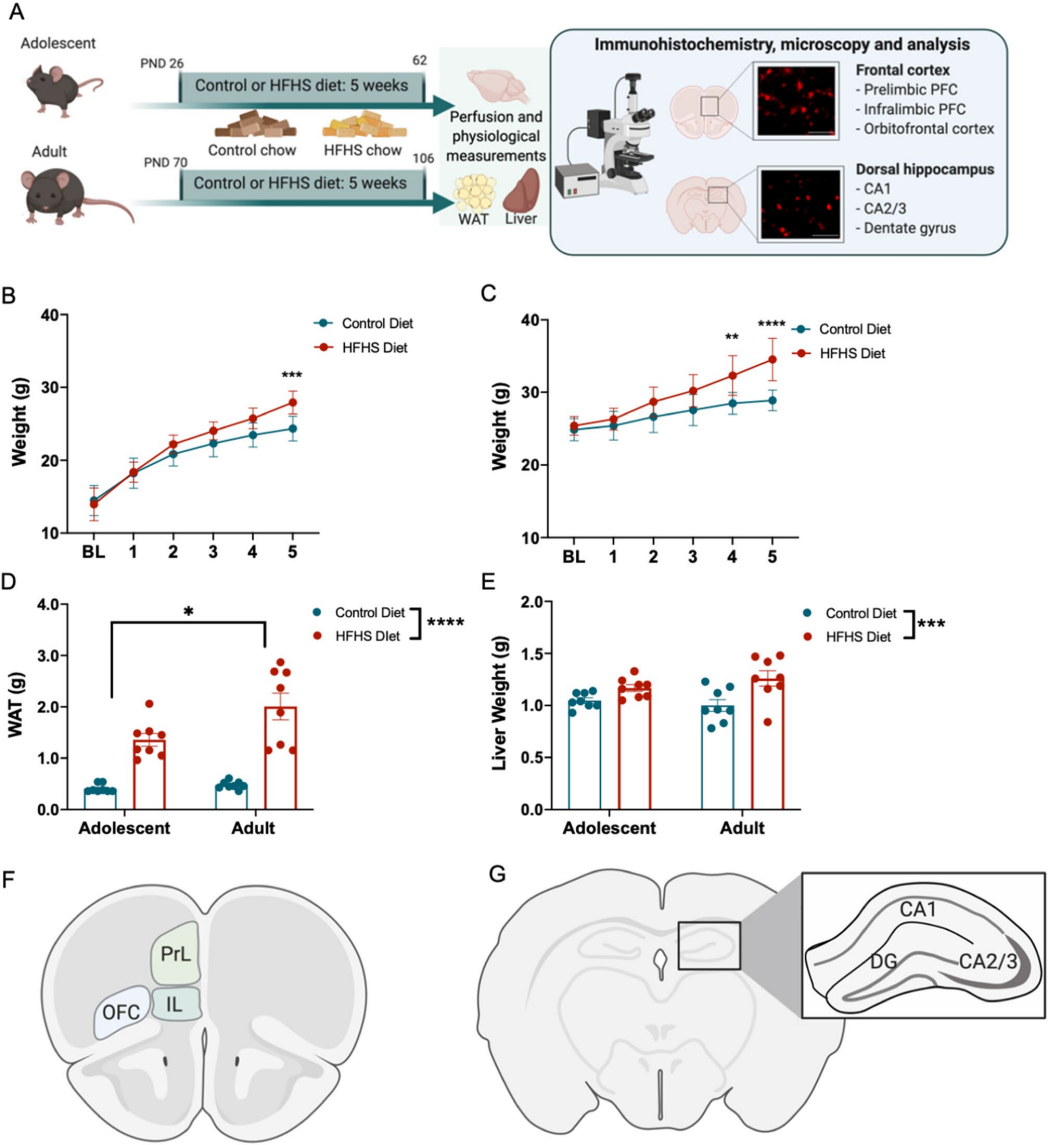
Physiological consequences of high-fat/high-sugar (HFHS) diet in male mice. (**A**) Timeline of the experimental procedure including diet administration and immunohistochemistry. Effect of HFHS or control diet on body weight in mice who commenced experimental diet manipulation across (**B**) adolescence and (**C**) as adults (*n* = *8* male mice per experimental condition). Impact of HFHS diet on white adipose tissue mass (WAT) (**D**), and liver weight (**E**). Schematic representations of the brain regions of interest in the (**F**) prefrontal cortex (bregma + 2.1 mm) and (**G**) dorsal hippocampus (bregma -2.06 mm). Data were assessed using repeated measures ANOVA and two-way ANOVAs *p < 0.05, **p < 0.01, ***p < 0.001, ****p < 0.0001. Panels (**A**,**F**,**G**) created with BioRender.com.

Mice were exposed to either a control diet or a HFHS diet (Fig. 1A), and mice in all age and diet groups gained weight over the 5 week experimental period, (Fig. 1B,C; adolescent F(5,70) = 321.2, *p* < 0.0001, adult F(5,70) = 155.4, *p* < 0.0001). Consistent with previous observations^6^, mice that consumed a HFHS diet across adolescence showed partial protection to weight gain (diet × time: F(5,70) = 8.904, *p* < 0.0001, diet group differences from week 5 onwards, *p* = 0.0004). Adult mice that consumed a HFHS diet gained significantly more weight than controls (diet × time: F (5,70) = 21.72, *p* < 0.0001 with group differences shown from week 4 (*p* = 0.0016). The HFHS diet also increased adiposity measured by retroperitoneal and gonadal white adipose tissue mass (WAT, Fig. 1D, main effect diet F(1,28) = 71.65, *p* < 0.0001) and liver weights (Fig. 1E, main effect diet F(1,28) = 13.84, *p* < 0.001).

### Development of PV+ neurons and PNN populations in the prefrontal cortex across age groups

**Figure 2:**
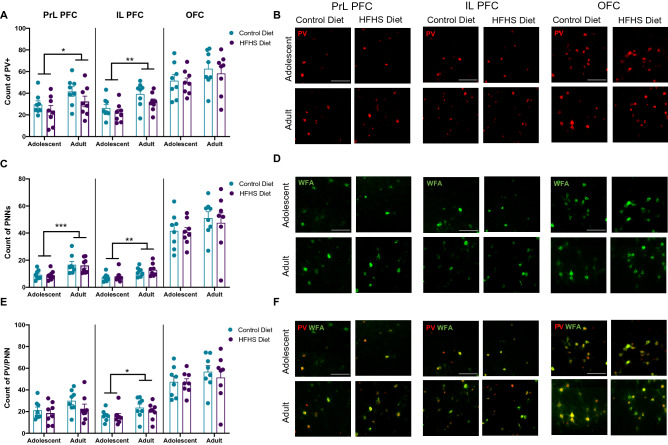
Developmental changes to PV and PNN populations within frontal cortical subregions. Mice in the adult cohort had significantly greater numbers of (**A**,**B**) PV+ neurons and (**C**,**D**) PNNs in the PrL and IL PFC, however no age differences were observed in the OFC. There were significantly more PV/PNN colocalised neurons in the adult cohort (**E**,**F**) in the IL. Data were assessed using two-way ANOVAs *p < 0.05, **p < 0.01. Representative photomicrographs were taken at 40X magnification (red—parvalbumin, green—WFA + PNN), scale bars show 100 μm.

Age-dependent increases in PV+ neurons were observed in the PrL (F(1,28) = 5.356, *p* = 0.028) and IL (F(1,28) = 11.55, *p* = 0.002) but not the OFC (F(1,28) = 2.900. *p* = 0.100), as shown in Fig. 2A,B. No group effects of diet was found in PrL (F(1,28) = 2.818, *p* = 0.104, diet × age: F(1,28) = 0.1661, *p* = 0.687), IL (F(1,28) = 3.132, *p* = 0.088, diet × age: F(1,28) = 0.213, *p* = 0.648) or OFC (F(1,28) = 0.272, *p* = 0.606, diet × age: F(1,28) = 0.071, *p* = 0.792).

The increase in PV+ neurons in adult animals compared to the adolescent group corresponded with increased WFA + PNN numbers in the adult group, who had more PNNs in the PrL (F(1,28) = 17.23, *p* = 0.0003) and IL (F(1,28) = 11.75, *p* = 0.002) but not OFC (F(1,28) = 2.46, *p* = 0.128) as shown in Fig. 2C,D. No main effects of diet on PNN counts were observed in the PrL (F(1,28) = 0.070, *p* = 0.793, diet × age: F(1,28) = 0.004, *p* = 0.949), IL (F(1,28) = 0.635, *p* = 0.432, diet × age: F(1,28) = 0.066, *p* = 0.799), or OFC (F(1,28) = 0.195, *p* = 0.662, diet × age: F(1,28) = 0.058, *p* = 0.811).

The adult group had greater PV/PNN colocalisation in the IL (F(1,28) = 4.657, *p* = 0.04), but not in the PrL (F(1,28) = 3.216, *p* = 0.084) or OFC (F(1,28) = 1.577, *p* = 0.22), as shown in Fig. 2E,F. HFHS diet had no group effects on PV/PNN colocalisation in the PrL (F(1, 28) = 1.917, *p* = 0.177, diet × age: PrL (F(1, 28) = 0.451, *p* = 0.507), IL F(1,28) = 0.635, *p* = 0.432, diet × age: F(1,28) = 0.609, *p* = 0.442) or OFC (F(1,28) = 0.294, *p* = 0.59, diet × age: F(1,28) = 0.173, *p* = 0.681).

Next, we examined whether associations were present between WAT mass and PFC regional PV and PNN markers in each age group. We found no significant correlations between WAT and PV+ neurons in the PrL in both adolescent and adult cohorts (Fig. S1A, adolescent *p* = 0.206, adult *p* = 0.484), IL (Fig. S1B, adolescent *p* = 0.176, adult *p* = 0.14) and OFC (Fig. S1C, adolescent *p* = 0.374, adult *p* = 0.191); WAT and PNN counts in the PrL (Fig. S1D adolescent *p* = 0.487, adult *p* = 0.393), IL (Fig. S1E adolescent *p* = 0.399, adult *p* = 0.485) and OFC (Fig. S1F, adolescent *p* = 0.431, adult *p* = 0.165); or WAT and PV/PNN in the PrL (Fig. S1G adolescent, *p* = 0.295, adult *p* = 0.447), IL (Fig. S1H, adolescent *p* = 0.384, adult *p* = 0.196) and OFC (Fig. S1I adolescent *p* = 0.408, adult *p* = 0.166).

### Adult HFHS diet decreases PV+ neurons and PNNs in the hippocampus

**Figure 3:**
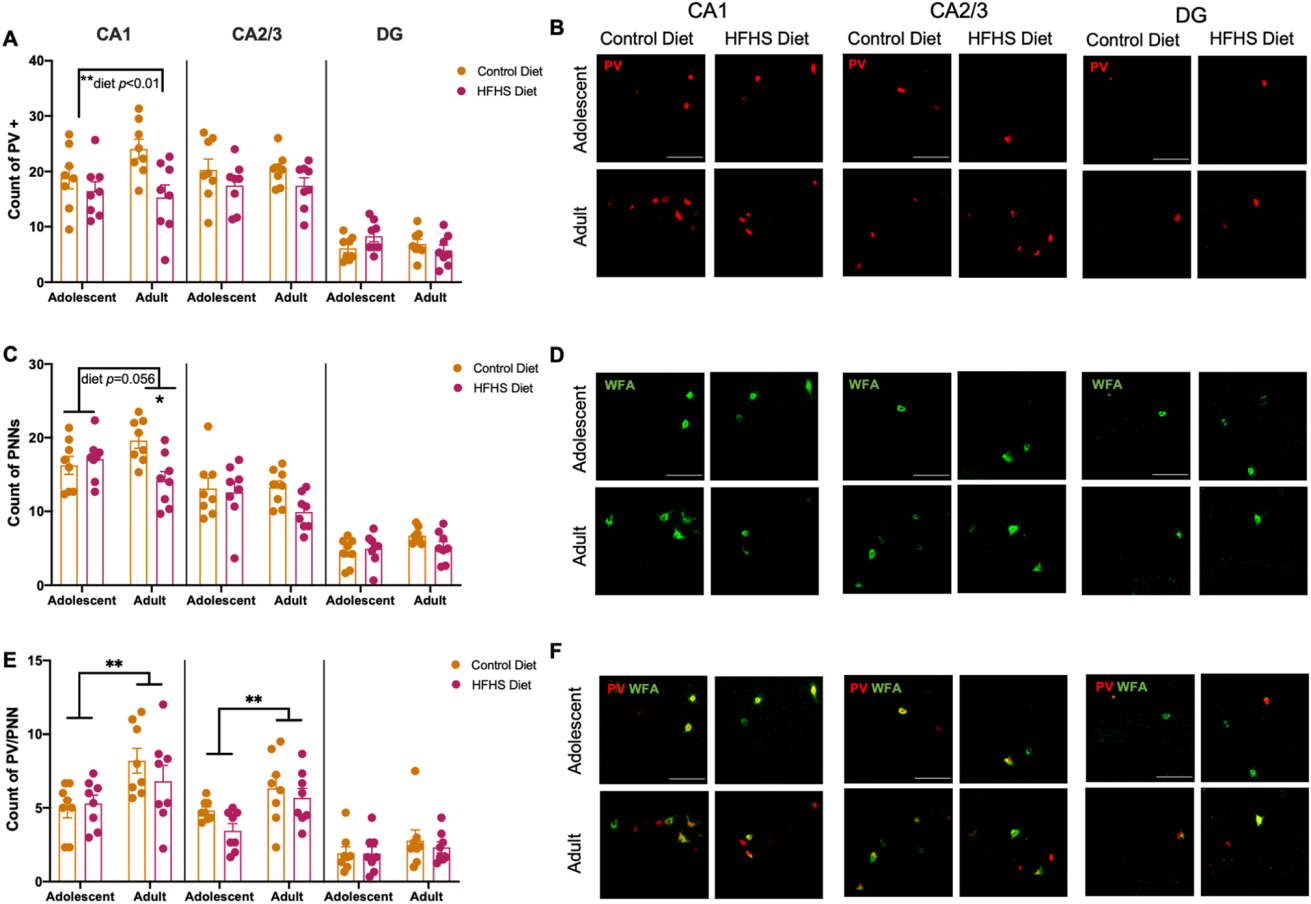
Age and HFHS diet effects on hippocampal subregions. Mice that consumed HFHS diet had fewer (**A**,**B**) PV+ neurons in CA1, but not CA2/3 or DG. Adult mice that consumed HFHS diet had fewer PNNs in the CA1, however no group differences were seen in the CA3 or DG (**C**,**D**). There were significantly more PV/PNN colocalised neurons in the adult cohort (**E**,**F**) in the CA1 and CA2/3, but not DG. Data were assessed using two-way ANOVAs *p < 0.05, **p < 0.01. Representative photomicrographs were taken at 40X magnification (red—parvalbumin, green—WFA + PNN), scale bars show 100 μm.

Mice that consumed HFHS diet had fewer PV+ neurons in the CA1 (Fig. 3A,B; main effect diet F(1,28) = 8.291, *p* = 0.008) but not the CA2/3 (F(1,28) = 3.474, *p* = 0.073) or DG (F(1,28) = 0.3032, *p* = 0.586). Hippocampal PV+ neurons did not differ between age groups in the CA1 (F(1,28) = 1.076, *p* = 0.308, diet × age: F(1,28) = 2.686, *p* = 0.112), CA2/3 (F < 1, diet × age: F(1,28) = 0.0004; *p* = 0.982) or DG (F(1,28) = 0.988; *p* = 0.329, diet × age F(1,28) = 3.563, *p* = 0.069).

As shown in Fig. 3C,D, adult mice that consumed HFHS diet had fewer PNNs in the CA1 (diet × age: F(1, 28) = 7.660, *p* = 0.01, significant simple main effect of diet in adult mice *p* = 0.002, but not adolescent *p* = 0.596). No group differences were seen in the CA3 (diet × age: F(1,28) = 1.419, *p* = 0.244) or DG (F(1,28) = 2.203, *p* = 0.149).

Age effects were observed on the colocalisation of PV+ neurons and PNNs (Fig. 3 E,F). Adult mice had more PV+ neurons surrounded by PNNs in the CA1 (F(1,28) = 9.003, *p* = 0.006) and CA2/3 (F(1, 28) = 9.973, *p* = 0.004) but not DG (F(1, 28) = 1.566, *p* = 0.22). No effect of HFHS diet was observed in the CA1 (diet F(1,28) = 0.424, *p* = 0.52, diet × age: F(1,28) = 1.199, *p* = 0.283), CA2/3 (diet F(1,28) = 2.92, *p* = 0.099, diet × age: F(1, 28) = 0.367, *p* = 0.549), or DG (diet F(1,28) = 0.228, *p* = 0.637, diet × age: F(1,28) = 0.190. *p* = 0.666).

We examined whether associations were present between WAT mass and hippocampal regional PV and PNN markers in each age group using linear regressions. We found a significant correlation between WAT mass and PV+ neurons in the CA2/3 of the adolescent, but not adult cohort (Fig. S2B adolescent, *p* = 0.031, adult, *p* = 0.431), however no further correlations were seen in in the CA1 (Fig. S2A adolescent, *p* = 0.078, adult, *p* = 0.125), and DG (Fig. S2C adolescent *p* = 0.104, adult *p* = 0.134). Significant associations were observed between WAT and PNN counts in the adult cohort in the CA1 (Fig. S2D adolescent *p* = 0.25, adult *p* = 0.024) and CA2/3 (Fig. S2E adolescent *p* = 0.445 , adult *p* = 0.015), but not the DG (Fig. S2F adolescent *p* = 0.249, adult *p* = 0.067); and no significant correlations between WAT and PV/PNN in adult and adolescent cohort in the CA1 (Fig. S2G adolescent *p* = 0.319, adult *p* = 0.452), CA2/3 (Fig. S2H adolescent *p* = 0.006 , adult *p* = 0.481) and DG (Fig. S2I adolescent *p* = 0.424, adult *p* = 0.394).

### HFHS diet consumption changed PFC microglia density and morphology

**Figure 4:**
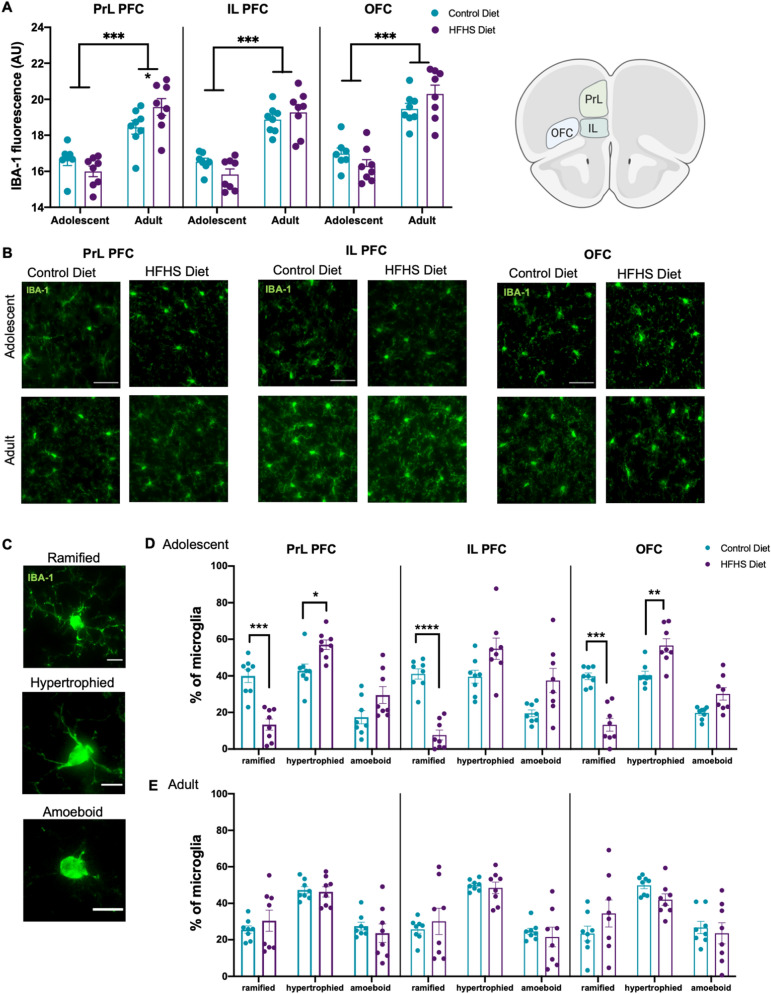
HFHS diet-evoked changes to PFC microglia density and morphology. Mice in the adult cohort had a significantly greater (**A**,**B**) IBA-1 fluorescence in all three prefrontal subregions. Consumption of the HFHS diet evoked a selective increase (**A**,**B**) in IBA-1 density in the PrL cortex in the adult cohort. Mice that consumed HFHS diet across adolescence had proportionally fewer (**C**,**D**) ramified ‘resting’ microglia in all three regions and more hypertrophied ‘active’ microglia in the PrL and OFC. In contrast, no diet-related changes (**C**,**E**) in PFC microglia morphology was seen in mice that consumed HFHS diet as adults. Data were assessed using two-way ANOVAs and *p < 0.05, **p < 0.01, ***p < 0.001, ****p < 0.0001. Representative photomicrographs were taken at 40X magnification (green—IBA-1 + microglia), scale bars show 50 μm for IBA-1 + density and 10 μm for IBA-1 + morphology.

Microglia undergo extensive morphological restructuring in response to exogenous and endogenous environmental events that may underlie central nervous system dysfunction^52^. Microglia were immunostained with ionized calcium binding adaptor molecule-1 (IBA-1) and fluorescent intensity and morphology was assessed. Overall, adult mice had greater PFC microglial density compared to adolescents (Fig. 4A,B, main effects of age PrL: F(1,27) = 51.07, *p* < 0.001, IL: F(1,27) = 83.18, *p* < 0.001, OFC: F(1,27) = 75.08, *p* < 0.001). HFHS diet evoked changes to microglia density that differed across age groups—HFHS diet increased IBA-1 density in adult but not adolescent mice in the PrL (diet × age: F(1,27) = 6.91, *p* = 0.025, simple main effect of diet the adult group F(1,27) = 4.704, *p* = 0.039, but not adolescent F(1,27) = 1.462, *p* = 0.237), but not in the IL (diet × age: F(1,27) = 3.0, *p* = 0.095) and OFC (diet × age: F(1,27) = 4.059, *p* = 0.054).

Microglia can be visually classified using morphological characteristics that align to functional phenotypes^47^. As such, microglia were classified into one of three morphological states: (a) *ramified* microglia were defined as having multiple radially projecting processes and an oval cell body, (b) *hypertrophied* microglia, which were classified by their enlarged darkened soma and shorter, thicker, and less branched processes, and (c) *amoeboid* microglia, where are defined as having densely stained, enlarged cell bodies with few short, if any, processes (Fig. 4C). Hypertrophied and amoeboid morphologies reflect activated states often seen in obesity^53^, whereas ramification reflects a resting, or surveillance state.

Using these classifications, we observed significant effects of HFHS diet consumed across adolescence on the morphology of microglia (diet × morphology—PrL: F (2,28) = 13.94, *p* < 0.0001; IL: F(2,28) = 15.51, *p* < 0.0001; OFC: F(2,28) = 23.07, *p* < 0.0001, Fig. 4D). Post hoc analyses revealed that mice that consumed HFHS diet across adolescence had proportionally fewer ramified ‘resting’ microglia (PrL *p* = 0.0002, IL *p* < 0.0001, OFC *p* = 0.0001), and more hypertrophied ‘active’ microglia (PrL *p* = 0.021, OFC *p* = 0.008) than controls. In contrast, no diet differences in PFC microglia morphology was seen in in adult mice (diet × morphology PrL F(2, 28) = 0.496, *p* = 0.615; IL F(2, 28) = 0.31, *p* = 0.736; OFC F(2, 28) = 1.478, *p* = 0.245, Fig. 4E).

We examined whether associations were present between WAT mass and prefrontal microglial density and morphology in each age group using linear regressions. We found significant correlations between IBA-1 density and WAT mass in the adult cohorts in the PrL (Fig. S3A adolescent *p* = 0.198, adult *p* = 0.042), OFC (Fig. S3C adolescent *p* = 0.288, adult *p* = 0.016), but not IL (Fig. S3B adolescent *p* = 0.134, adult *p* = 0.148). We found significant correlations between hypertrophied ‘active’ microglia and WAT mass in both the adolescent and adult cohorts in the PrL (Fig. S3D adolescent *p* = 0.004, adult *p* = 0.293), IL (Fig. S3E adolescent *p* = 0.007, adult *p* = 0.17) and OFC (Fig. S3F adolescent *p* < 0.001, adult *p* = 0.041).

### HFHS diet consumption resulted in changes to hippocampal microglia density and morphology

**Figure 5:**
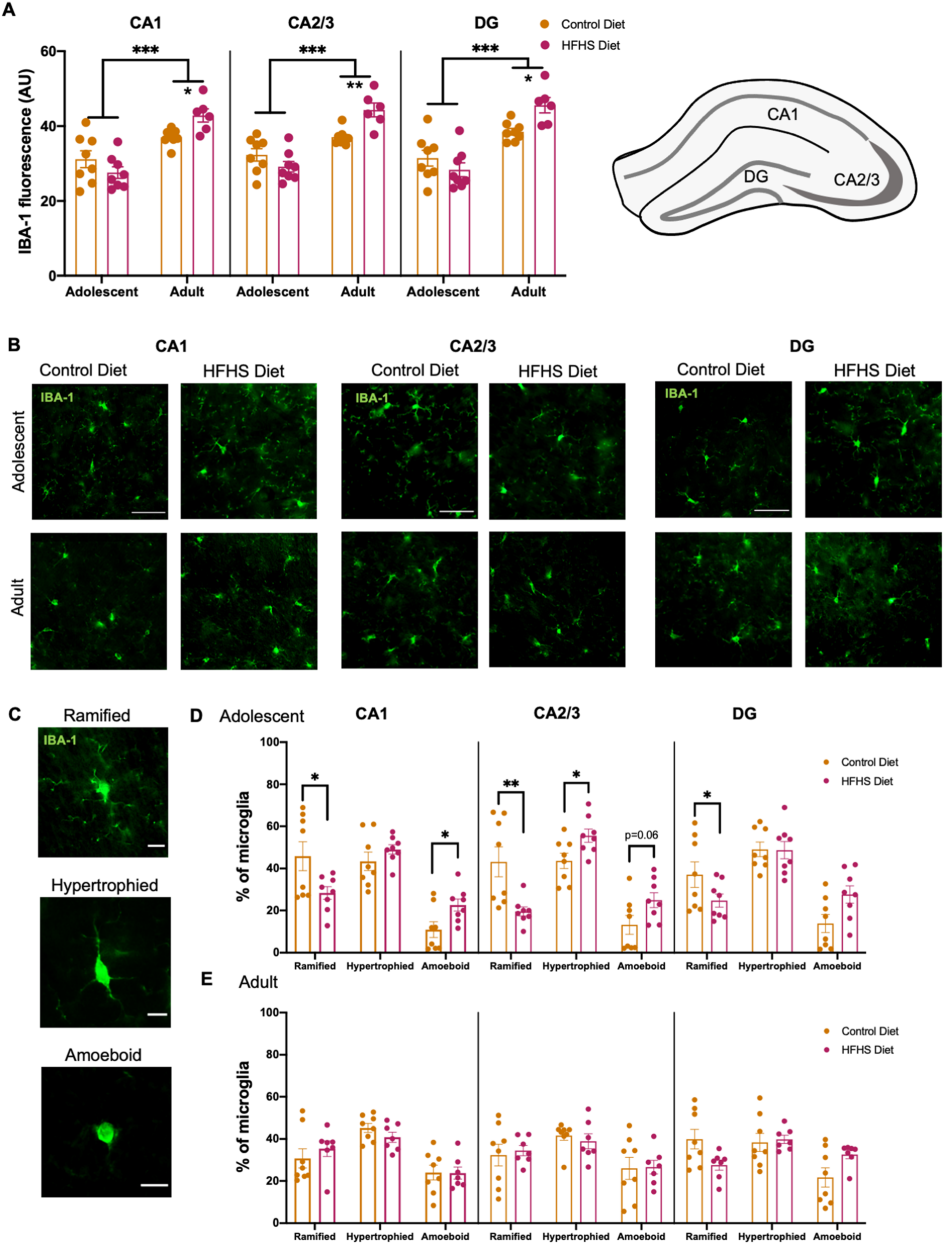
HFHS diet changes to hippocampal microglia density and morphology. Mice in the adult cohort had a significantly greater hippocampal IBA-1 fluorescence and adult mice that consumed the HFHS diet had significantly greater IBA-1 fluorescence compared to control diet mice (**A**,**B**). Mice that consumed HFHS diet across adolescence had proportionally fewer ramified ‘resting’ microglia and greater activated phenotypes (hypertrophied and amoeboid) in all three hippocampal subregions (**C**,**D**). In contrast, no diet-related changes (**C**,**E**) in microglia morphology were seen in mice that consumed HFHS diet as adults. Data were assessed using two-way ANOVAs and *p < 0.05, **p < 0.01, ***p < 0.001. Representative photomicrographs were taken at 40X magnification (green—IBA-1 + microglia), scale bars show 50 μm for IBA-1 + density and 10 μm for microglia morphology.

As shown in Fig. 5A,B, adult HFHS diet mice had a significantly greater IBA-1 fluorescence than control diet mice in the CA1 (F(1,26) = 5.58, *p* = 0.026), CA2/3 (F(1,26) = 11.9, *p* = 0.002) and DG (F(1,26) = 7.31, *p* = 0.012). As also seen in the PFC, the adult cohort had a greater hippocampal IBA-1 fluorescence than the adolescent group (CA1 F(1,26) = 39.43, *p* < 0.001, CA2/3 F(1,26) = 48.11, *p* < 0.001 and DG F(1,26) = 46.66, *p* < 0.001). Significant diet × age interactions were observed in the CA1 (F(1,26) = 7.800, *p* = 0.01), CA2/3 (F(1,26) = 13.16, *p* = 0.001), and DG (F(1,26) = 8.141, *p* = 0.008).

HFHS diet consumption altered hippocampal microglia morphology (Fig. 5C) in the adolescent (Fig. 5D) but not the adult mice (Fig. 5E): Adolescent morphology × diet interactions—CA1 F(2, 28) = 5.56, *p* = 0.009; CA2/3 F(2, 28) = 7.51, *p* = 0.002; DG F(2, 28) = 3.60, *p* = 0.04; Adult morphology × diet interactions—CA1 F(2, 26) = 0.74, *p* = 0.486; CA2/3 F(2,26) = 0.252, *p* = 0.779; DG F(2,26) = 2.372, *p* = 0.115).

We examined whether correlations were present between WAT mass and hippocampal IBA-1 fluorescence or hypertrophied in each age group using linear regressions. We found significant correlations between IBA-1 fluorescence and WAT mass in the adult and adolescent cohorts in the CA1 (Fig. S4A adolescent, *p* = 0.092, adult, *p* < 0.0001), CA2/3 (Fig. S4B adolescent, *p* = 0.046, adult, *p* < 0.0001), and DG (Fig. S4C adolescent, *p* = 0.107, adult, *p* < 0.0001).

Furthermore, we found a significant correlation between the hypertrophied ‘active’ morphology and WAT mass in the CA1 in the adult but not the adolescent cohort (Fig. S4D adolescent, *p* = 0.4, adult *p* = 0.026), in the adolescent but not adult CA2/3, (Fig. S4E adolescent *p* = 0.019, adult *p* = 0.486), and no correlations in either age group in the DG (Fig. S4F adolescent *p* = 0.48, adult *p* = 0.297).

### IBA-1 fluorescence in the PrL PFC was associated with decreased numbers of PV neurons and PNNs in adult mice

We examined whether associations were present between IBA-1 fluorescence and PFC PV and PNN markers in each age group using linear regressions. We found a significant correlation between IBA-1 fluorescence and PV neuron counts in the PrL in the adults but not adolescent cohort (Fig. S5A adolescent *p* = 0.498, adult *p* = 0.048), however no further significant correlations were seen in the IL (Fig. S5B adolescent *p* = 0.180, adult *p* = 0.414) and OFC (Fig. S5C adolescent *p* = 0.408, adult *p* = 0.147). Additionally, a significant correlation between IBA-1 fluorescence and PNN count was found in the PrL in the adult, but not adolescent cohort (Fig. S5D adolescent *p* = 0.798, adult *p* = 0.023). No significant correlations were found in the IL (Fig. S5E adolescent *p* = 0.106, adult *p* = 0.130) and OFC (Fig. S5F adolescent *p* = 0.568, adult *p* = 0.415). Furthermore, no significant correlations between IBA-1 fluorescence and PV/PNN count were found in the PrL (Fig. S5G adolescent *p* = 0.319, adult *p* = 0.06), IL (Fig. S5H adolescent *p* = 0.314, adult *p* = 0.473), and OFC (Fig. S5I adolescent *p* = 0.337, adult *p* = 0.153).

### No significant associations between IBA-1 fluorescence and PV neurons and PNNs in the dorsal hippocampus

Using linear regressions, we examined whether associations were present between IBA-1 fluorescence and hippocampal PV and PNN markers in each age cohort. No significant correlations were found between IBA-1 fluorescence and PV counts in CA1 (Fig. S6A adolescent *p* = 0.428, adult *p* = 0.185), CA2/3 (Fig. S6B adolescent *p* = 0.101, adult *p* = 0.291), and DG (Fig. S6C, adolescent *p* = 0.476, adult *p* = 0.183). Additionally, no significant correlations were seen between IBA-1 fluorescence and PNN counts in CA1 (Fig. S6D adolescent *p* = 0.540, adult *p* = 0.290), CA2/3 (Fig. S6E, adolescent *p* = 0.640, adult *p* = 0.425), or DG (Fig. S6F, adolescent *p* = 0.313, adult *p* = 0.530). Finally, no significant correlations were found between IBA-1 fluorescence and PV/PNN counts in the CA1 (Fig. S6G, adolescent *p* = 0.376, adult *p* = 0.234), CA2/3 (Fig. S6H, adolescent *p* = 0.470, adult *p* = 0.376), and DG (Fig. S6I, adolescent *p* = 0.212, adult *p* = 0.420).

## Discussion

Here, we show that consumption of a HFHS diet for 5 weeks commencing in either adolescence (P26) or adulthood (P68) had age- and region-specific effects on PV neurons, PNNs and microglia within regions of the murine PFC and hippocampus. Moreover, our immunohistochemical examination demonstrated that populations of PV neurons and PNNs increased with age in the mPFC, but did not change in the hippocampus.

Our observation that PV-expressing neurons and their surrounding PNNs have delayed developmental trajectories in the medial PFC, but not the hippocampus, supports previous studies that showed PNN development in the medial PFC extends through adolescence^17,54–56^. We observed increased PV and PNN counts in the PrL and IL PFC in the adult cohort compared to the adolescent cohort of mice. Our results contrast aspects of previous studies that found significant increases in PV and PNN counts in the PrL and IL PFC between juvenile (P24) and adolescent rats (P35), but no significant differences between adolescent and adult rats (P70)^17^. However, the increases in PV and PNNs in the PrL and IL with increased age observed in our experiment indicate that there is a more enduring age-dependent refinement within the mouse medial PFC, which may extend further into adulthood than previously observed^17^. Moreover, we extended our histological examination to encompass the OFC, another frontal cortical region that is involved in behavioural regulation, where we did not see differences in PV and PNN counts between the age groups, which indicates that populations of PV neurons and PNNs in the OFC reach maturity before the PrL and IL PFC.

An explanation for the increase in PV immunoreactivity between our age groups of mice may be a developmental increase in the expression of PV-protein in fast-spiking interneurons, which is reflected by increased PV-immunoreactivity and mRNA during the process of brain maturation^21,22^. Of note, we also found that colocalisation of PNNs with PV neurons in the IL PFC was greater in adult than adolescent animals. This indicates that the IL PFC likely has the latest critical window of maturation in mice, as the envelopment of PV neurons with PNNs is widely acknowledged to be a marker of the closure of critical windows of plasticity in neurodevelopment. Although the exact molecular orchestration of the development of the PFC is still largely unknown, behavioural studies functionally demonstrate that the IL PFC is implicated in mood regulation and fear memory extinction, behaviours that typically develop in late adolescence^57,58^. This functional observation combined with our cytoarchitectural findings indicates that the IL PFC is likely one of the last frontal cortical regions to reach maturity. Studies that have examined human post-mortem tissue samples of the frontal cortices and hippocampus have also shown that PV neurons are present from birth, although at low levels, and increase to peak levels at 2 years of age^59^. PNNs begin to form as early as the second month of life but do not reach a mature appearance until around 8 years of age^59^. Interestingly, longitudinal proteomic and transcriptomic data from human dorsolateral PFC samples show a dynamic reduction in the expression of neurocan, a lectican that forms glycosaminoglyan side chains of PNNs after birth^60^. Aggrecan, another lectican, increases in expression across development and into adulthood, suggesting that it is continuously used in the remodelling of PNNs^59^.

The envelopment of PV neurons with PNNs not only marks the closure of developmental windows of heightened neuroplasticity, but also provides a protective microenvironment around neurons^37^. Neurons without PNNs have increased sensitivity to environmental and endogenous toxins, which can lead to functional dysregulation^33,34,36^. The biomolecular mechanisms underpinning the endogenous modulation of PNN integrity are yet to be fully elucidated. Possible mediators include extracellular proteolytic enzymes called matrix metalloproteinases (MMPs) that are secreted by neurons and glia. Activated MMPs cleave structural components of PNNs, and so permit the dynamic remodelling of synapses and alterations to neuronal plasticity^48,61^. However, the upregulation of MMP activity is seen in states of neuroinflammation and neurodegenerative diseases^49^, where MMPs secreted by microglia could aberrantly cleave PNNs and expose neurons to toxins.

Interestingly, we did not see effects of HFHS diet on PV neuron immunoreactivity, PNNs or their colocalisations in regions of the PFC. Previous studies have shown that intermittent daily access to a HFHS diet in rats, which causes binge-like behaviours, reduced PV neuron immunoreactivity and PNNs in the PFC^24,26^, and high fat diets (> 60% fat) have been related to reduced PNNs in the PrL and orbitofrontal cortex in male rats^24^. This finding suggests that diet formulations and administration protocols may have differential effects on PFC PV and PNNs, and also supports studies that have observed distinct cross-species expression patterns of PNNs between mice and rats^62^.

In contrast to the PFC, we saw deleterious effects of HFHS diet on populations of PV neurons and PNNs in the hippocampus, particularly in adult mice. Diet-associated reductions of PV neuron immunoreactivity and PNNs were observed within the CA1 of the adult cohort, suggesting that this region may be more vulnerable to nutritional stress. This aligns with previous observations of pronounced obesity-induced cognitive deficits in spatial memory^14,15,63,64^, as PV-mediated inhibitory neurotransmission within the CA1 is required for the stabilisation of place cells^65^. The most prominent diet-evoked changes to PV neurons and PNNs were observed in the adult cohort, supported further by negative associations between adiposity and numbers of PNNs within the CA1 and CA2/3 in the adult cohort. Thus, PV neurons and PNNs in the younger brain may have some resilience to HFHS diet-induced dysregulation within the hippocampus. This may be because of increased remodelling of PNNs during early life which would allow for the protective microenvironment to be repaired more efficiently.

Our observation of changes to PNNs and PV neuron populations in the hippocampus align with studies showing that hippocampal function is vulnerable to the negative functional consequences of HFHS diet and obesity^8,12,14–16^. Obesity is associated with an increase in circulating inflammatory cytokines, which are generated by enlarged and increased populations of adipocytes in white adipose tissue. Increased inflammation leads to disruption of tight junction proteins such as claudin-5 and occludin within the blood brain barrier (BBB)^66^. The increased permeability of the BBB allows the influx of leukocytes, and engages microglia to produce pro-inflammatory cytokines^67^. These mechanisms are proposed to underlie both the onset of microgliosis and have further downstream damage to neuronal function.

Mice that consumed HFHS as adults showed increased microgliosis in the PrL PFC and all hippocampal subregions measured by IBA-1 fluorescence compared to the adolescent cohort, which was also associated with increased adiposity. Given that microglia respond to cytokines, this observation suggests that there may be some resistance to HFHS-induced neuroinflammation in younger animals, which has been previously observed in rats^44^. However, when we examined morphological changes in microglia—from resting (ramified) to active (hypertrophied) states—we found a significant reduction in ramified microglia and related increases in hypertrophied microglia within the PFC of mice exposed to the HFHS-diet during adolescence, but not as adults, which was positively correlated with adiposity. The switch between microglial ramification to reactive phenotypes in the PFC is associated with alterations in neuronal activity^74^, and may be linked to the more pronounced functional changes in PFC-mediated cognitive tasks observed in mice fed obesogenic diets across adolescence^6^. We therefore suggest that the obesity-evoked activation of microglia could provide a neuroprotective mechanism in the adolescent brain; however, this may have enduring functional consequences on neuroplasticity. Ramified microglia are involved in the pruning of excess synapses during neurodevelopment^75,76^ and control of the neurogenic niche within the dentate gyrus^77^. As such, the activation of microglia by neuroinflammation may divert their role from the refinement of neuronal interactions and plasticity, leading to a derailment of neurodevelopmental trajectories.

However, the link between microgliosis and damage to or dysfunction of PV neurons is complex. We did not observe relationships between IBA-1 fluorescence and PV neurons/PNNs in the hippocampus, but did see a negative association between PV neurons/PNNs and IBA-1 fluorescence in the PrL PFC of adult mice. When microglia become activated, they can release more cytokines and can exacerbate neuroinflammation^69^. Due to their high energy demands, PV neurons are particularly susceptible to dysfunction under inflammatory or oxidative conditions^70–73^. Microglia activation is one indicator of a neuroinflammatory environment^45^, however further studies are needed to examine whether other chemical markers of inflammation or oxidative stress were increased.

Whether the hippocampus is vulnerable to nutritional stress in young mice is less clear—with previous studies showing exacerbation of memory deficits and neuropathological changes seen in diet administration from an early age^13,78^, but other studies in adult rats and mice showing profound and rapid HFHS-dependent memory deficits^14,15^. Developmentally, the maturation of the hippocampus is complete before the PFC, and in keeping with this developmental pattern we did not observe differences between PV neurons or PNN populations within dorsal hippocampal subregions of the two age groups. However, we did see significant increases in PV/PNN colocalisation between the two age groups within the CA1 and CA2/3, indicating that the refinement of PV-mediated plasticity in these region may continue into early adulthood^79^. Furthermore, the dentate gyrus is a key region of adult hippocampal neurogenesis, the disruption of which can lead to pronounced memory impairment^80^. It has previously been shown that high fat or high sugar diets decreased histological markers of neuroproliferation in the dentate gyrus^63,81,82^, indicating that even relatively small shifts in cellular populations in the dentate gyrus can have a profound impact on hippocampal function. Microglia activation is known to have a negative impact on hippocampal neurogenesis^77^ which may provide another mechanism by which hippocampal function is disrupted by HFHS diet consumption^82^.

In summary, these data demonstrate that HFHS-diet leads to disturbances to PV neurons, PNNs and microglia in an age- and regionally distinct manner, with effects on PV neuron and PNNs numbers being more pronounced within the adult hippocampus, and some resilience to microgliosis in the hippocampus of adolescent mice. Moreover, we observed protracted changes in PV cell density, PNNs and microglia in the PrL and IL PFC outside of the expected peri-adolescent period, suggesting that these cellular populations continue to develop in these regions. Further studies should investigate the sex-dependent effects of HFHS diets across development, whereby notable sex differences in the PFC has been observed in microglia morphology following immune challenges^83^, PNN numbers^84^, and in adult rats fed high fat diets^27^. While changes in PNNs and PV neurons have been reported in animal models and humans with neuropsychiatric conditions^54^, the functional significance of these changes remain to be delineated with respect to dietary challenges—including obesity. Further studies utilising proteomics as well as whole-transcriptome sequencing will provide more in-depth molecular characterisation of how brain regions are affected by different nutritional states and help pin-point developmental windows of vulnerability. Moreover, measurement of MMP activity in the brain will provide insight into how in obesity could be altering microglia activity and the integrity of PNNs. Critically, a better understanding of the functional relationship between microglia, neurons and PNNs during sensitive windows of neurodevelopment such as adolescence, during ageing and in neuropathological states will provide innovative therapeutic strategies to prevent cognitive decline.

## Methods

### Animals and housing

Male C57Bl/6 J mice (N = 32, Jackson Laboratories, US) arrived into the lab aged 3 weeks old (P21—adolescent, N = 16, n = 8/diet group) or 9 weeks old (P63—adult, N = 16, n = 8/diet group). Mice were housed in 28 × 18 cm plastic cages with wire tops in groups of 4 and were maintained on a 12 h/12 h reversed light–dark cycle (lights off at 09:00) in non-SPF conditions. Mice weighed three times a week across the experimental period. All procedures were conducted in accordance with guidelines of the Canadian Council of Animal Care and approved by the University of Western Ontario Animal Use Subcommittee (protocol 2017-031), and in compliance with ARRIVE guidelines.

### Diet administration

Mice were habituated to the housing conditions for 5 days before being allocated to their diet manipulation groups. Diets administration began when mice were either 26 days old (which encompasses adolescence^85^) or at 68 days old (adult) as shown in Fig. 1A. Mice in the control diet condition had ad libitum access to rodent chow (Teklad Envigo 7913, 18% protein rodent diet, 3.1 kcal/g, 5% fat, 5% fibre). Mice allocated to the HFHS diet had ad libitum access to a Western Diet (Bioserv F6724, 4.57 kcal/g, anhydrous milkfat 20%, cholesterol 0.2%, 17.7% protein, 21.0% fat, 49.2% carbohydrates) and water. Mice in the HFHS diet condition received this diet for a total of 5 weeks up until sacrifice. All mice had ad libitum access to water.

### Histology

#### Tissue preparation

Mice were anaesthetised with ketamine/xylazine in sterile saline (100/10 mg/kg) and perfused transcardially with PBS (0.01 M, pH 7.4) followed by 4% PFA in PBS. Livers, gonadal and retroperitoneal white adipose tissue (WAT) were excised and weighed as further physiological measures of dietary effects.

Brains were extracted and stored in 4% PFA for 24 h at 4 °C. After this, they were transferred to 20% sucrose in PBS for 24 h at 4 °C, before being frozen and sliced to 30 μm coronal sections using a cryostat (Leica CM1950S). Four serially adjacent sections were obtained from each brain and stored in cryoprotectant (50% PBS, 25% ethylene glycol, 25% glycerol) at -20 °C. Dorsal hippocampal slices (bregma -1.6 to -2.4 mm) and frontal cortical slices containing prelimbic, infralimbic and orbitofrontal PFC (bregma + 2.3 to + 1.8 mm) were identified using a mouse brain atlas^86^ and selected based on their DAPI nuclear staining pattern.

### Immunohistochemistry

#### PV and PNN staining

Free floating sections were washed three times in PBS, permeabilised and blocked with 4% normal goat serum (NGS, S-1000, Vector Laboratories) and 0.1% bovine serum albumin (BSA, Sigma-Aldrich) in PBS + 0.1% Triton X-100 (PBS-T) solution for 2 h at room temp (~ 20 °C). Sections were incubated for 48 h at 4 °C with constant agitation in rabbit anti-PV antibody (1:2000, Abcam, ab11427) and PNNs were labelled with 488-fluorescein-conjugated *Wisteria floribunda* agglutinin (WFA) lectin (1:500; FL-1351 Vector Laboratories), which binds to the terminal N-acetylgalactosamine residues of CSPGs, these antibodies were diluted in 2% NGS and 0.1% BSA in PBS-T. The sections were then washed 3 × 10 min in PBS, followed by 2-h room temperature incubation in goat anti-rabbit AlexaFluor 594 secondary antibody (1:500, Invitrogen, A-11012). Sections were then washed three times in PBS and mounted onto SuperFrost + slides and coverslipped with antifade mounting medium (VectorShield hardset) with DAPI (4′,6-diamidino-2-phenylindole).

#### IBA-1 staining

IBA-1 immunoreactivity was used an indication of microglia activation^87^. Following blocking and permeabilisation, free floating sections were incubated for 48 h at 4 °C with constant agitation in rabbit anti-IBA-1 antibody (1:2000, Abcam, ab178846) in 2% NGS and 0.1% BSA in PBS-T, followed by 3 PBS washes and a 2 h room temperature incubation goat anti-rabbit AlexaFluor 488 secondary antibody (1:500, Invitrogen, A-11008). After three washes, sections were then mounted to slides and coverslipped as described above.

### Imaging

Immunostained sections were imaged using an EVOS FL digital cell imaging system (Thermofisher Scientific). Tiled images of three unilateral dorsal hippocampal sections and PFC sections per mouse per stain were obtained using a 40X dry objective and 405 nm, 470 nm or 585 nm LED light cubes (depending on the secondary antibody used for the staining protocol described above). The frontal cortex (bregma + 2.3 to + 1.8 mm) was divided into three subfields: Prelimbic (PrL) PFC, infralimbic (IL) PFC and orbitofrontal cortex (OFC) and the dorsal hippocampus (bregma -1.6 to -2.4 mm) was divided into the three subfields: CA1, CA2/3; and DG (Fig. 1F,G).

From the images, PNN counts, PV cell counts, and colocalised PV cells with PNNs were counted using Image-based Tool for Counting Nuclei plugin (Centre for Bio-image Informatics, UC Santa Barbara, CA, USA) for NIH ImageJ software (https://imagej.nih.gov/ij/). In the frontal cortices, counts were made from 500 μm × 500 μm areas taken from each subregion. In the dorsal hippocampus, subregional cell counts from the stratum pyramidal, stratum oriens and stratum radiatum of the CA1 and CA2/3, and the molecular, granular and polymorphic layers of the dentate gyrus, including the hilus were made based on anatomical characteristics referenced from The Mouse Brain in Stereotaxic Coordinates Atlas^86^.

For IBA-1 staining, 3 areas (240 μm × 190 μm) from each frontal cortex and hippocampal subregion were captured using 10 sequential z-stacks (1.2 μm) at maximum projection at 40X objective. ImageJ software was used to calculate fluorescence intensity of the IBA-1 stain, expressed as arbitrary units (AU) as has been previously published in diet studies^82^. This was conducted as an objective measure of the mean area of the IBA-1 stain in specific brain regions. As microglia can change shape dynamically within different environments, cell counts do not necessarily provide information regarding activation. Microglia morphology was determined based on visual inspection of the distal arborisation and cell body shape of IBA-1 positive cells based on categories—*ramified*, *hypertrophied* or *amoeboid* microglia, phenotypes that have been previously characterised in multiple studies^47,71,72,88,89^. We used a workflow to visually identify IBA-1 positive microglia phenotypes according to defined morphological characteristics^90^. Ramified microglia were identified as cells with small circular cell bodies and multiple (> 3) thin, highly branched processes. Hypertrophied microglia (also called reactive or primed microglia) had larger cell bodies that were oval or elongated in shape with > 2 branched, wide and enlarged processes. Amoeboid microglia had large cell bodies that had at most two short, rod-like processes without any branching or were completely devoid of any branches^47,88,89^. We coded the slides and a blinded independent observer then quantified them.

### Statistical analyses

All image analysis and cell counts were conducted by an experimenter who was blind to age and diet group allocations. Data are presented as mean ± SEM. Statistical analyses were carried out in SPSS Software (IBM SPSS Statistics for Windows, Version 24.0. Armonk, NY, USA) and GraphPad PRISM Software (Prism Software, version 8.0, Irvine, CA, USA) using repeated measures ANOVAs and two-way ANOVAs with Bonferroni or Fisher’s LSD post hoc tests, and data were screened for homoscedasticity by Levene’s test. Correlations were examined post hoc using one-tailed linear regressions and significance was set at *p* < 0.05.

## Supplementary Information

Supplementary information.

## Data Availability

Experimental data from these studies are available at https://osf.io/bh84n/?view_only=989dd78f2e2f42989d6f52291fa87f95.
