# Supplementary material for: Age-dependent and region-specific alteration of parvalbumin neurons, perineuronal nets and microglia in the mouse prefrontal cortex and hippocampus following obesogenic diet consumption
Source: Sci Rep. 2021 Mar 10;11:5593. doi: 10.1038/s41598-021-85092-x (PMC7970944; doi:10.1038/s41598-021-85092-x)
Supplement: Supplementary file 1 — Supplementary information. [file 41598_2021_85092_MOESM1_ESM.pdf]

Supplementary figures

Age-dependent and region-specific alteration of parvalbumin neurons, perineuronal nets and microglia in the mouse prefrontal cortex and hippocampus following obesogenic diet consumption

Amy C. Reichelt, Claire A. Lemieux, Oren Princz-Lebel, Ashmita Singh,

Timothy J. Bussey, Lisa M. Saksida

Supplementary figures

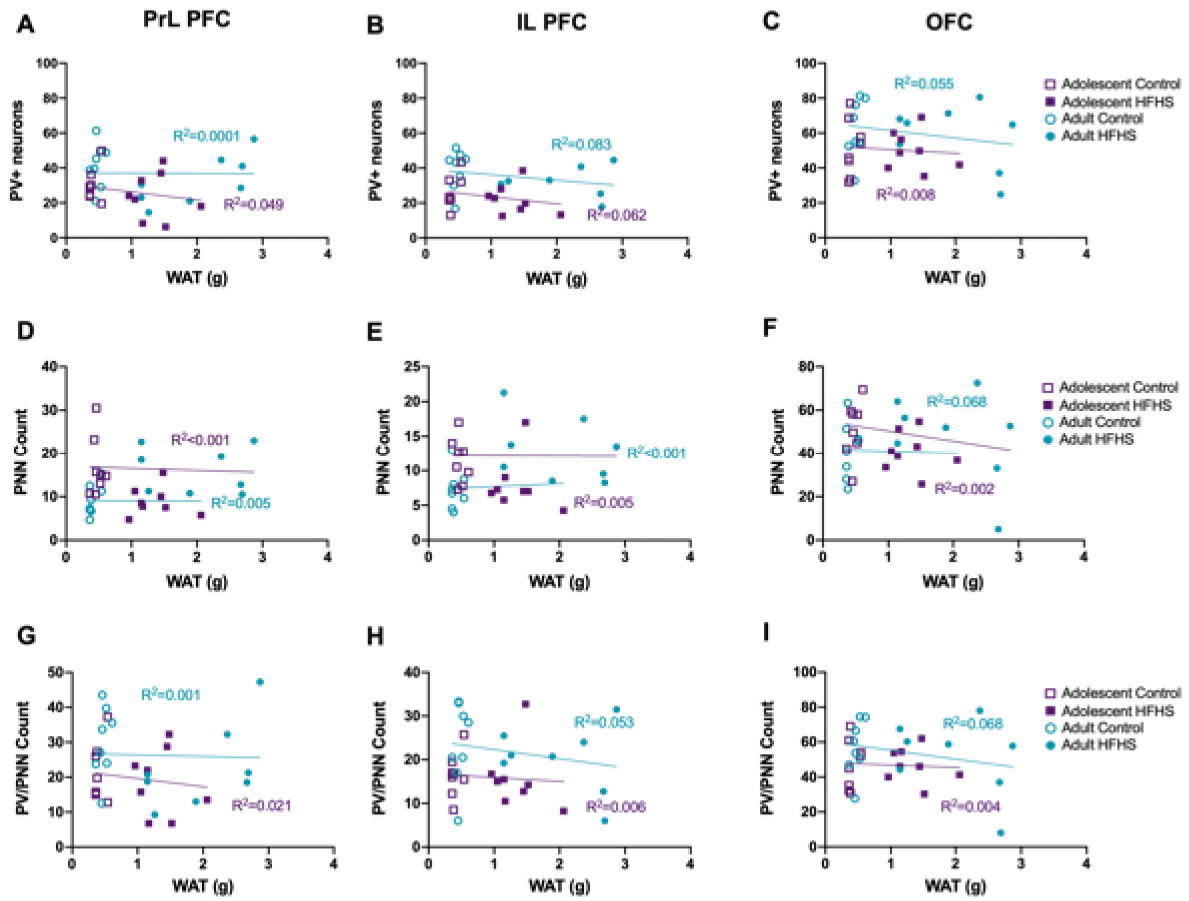

Figure S1. Linear regressions showing the association between PFC subregions (PrL, IL, OFC) PV+ neurons (A-C), WFA+ PNNs (D-F), PV/PNN colocalisation (G-I) and adiposity (white adipose tissue mass – WAT) in mice exposed to a HFHS diet across adolescence or as adults. R^2 values presented, *p<0.05, **p<0.01, ***p<0.001.

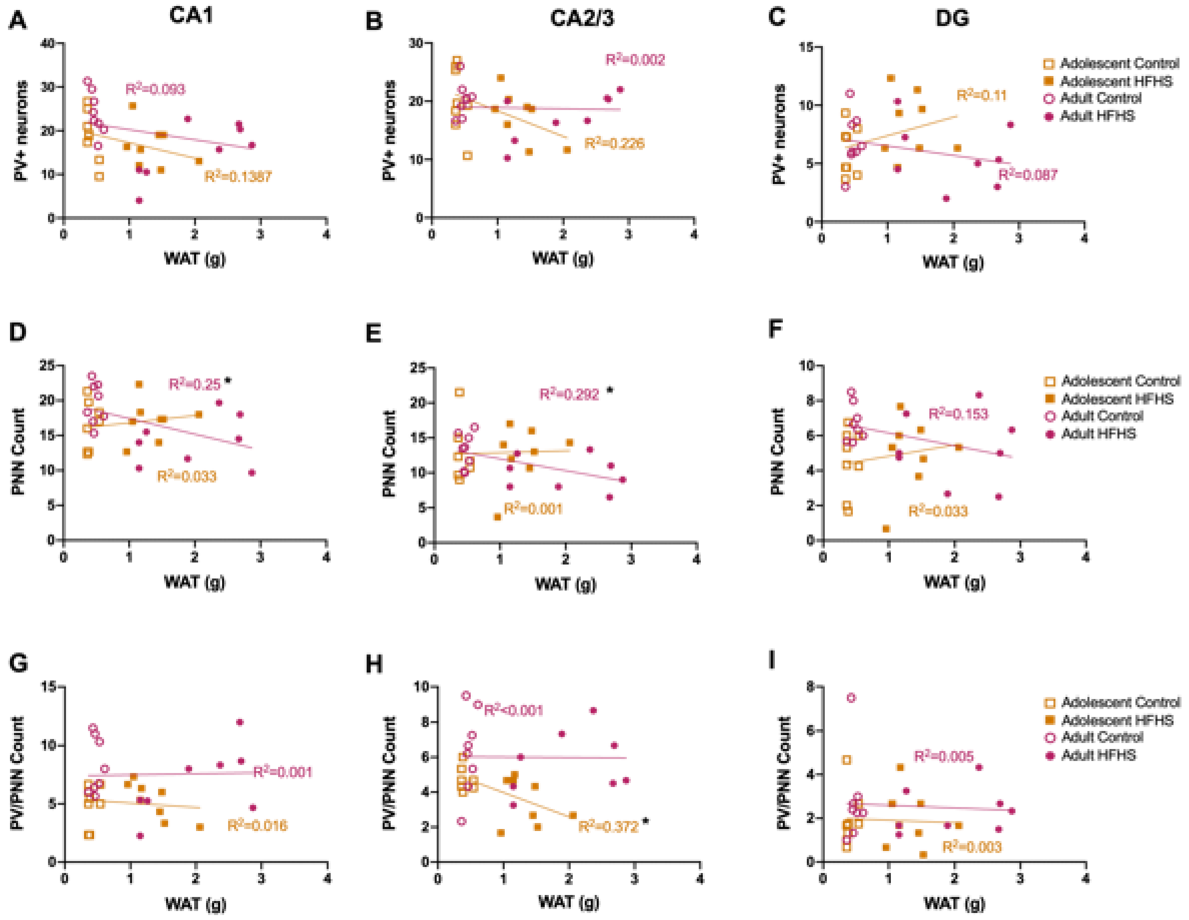

Figure S2 - Linear regressions showing the association between hippocampal subregions (CA1, CA2/3, DG) PV+ neurons (A-C), WFA+ PNNs (D-F) and PV/PNN colocalisation (G-I) and adiposity (white adipose tissue mass – WAT) in mice exposed to a HFHS diet across adolescence or as adults. R^2 values presented, *p<0.05, **p<0.01, ***p<0.001.

Supplementary figures

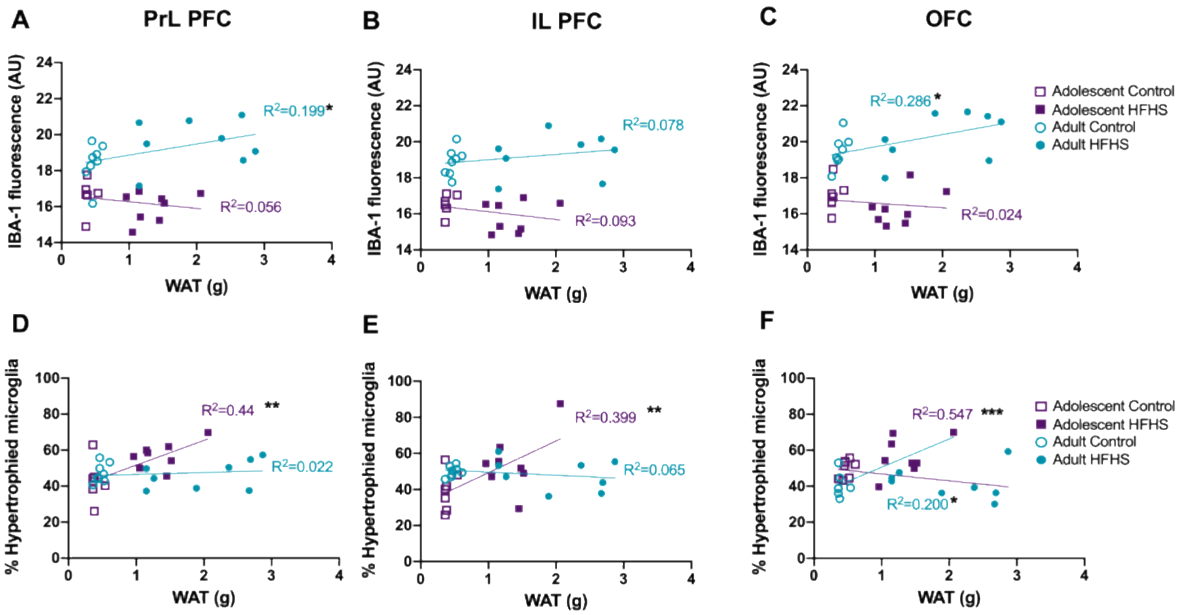

Figure S3 - Linear regressions showing the association between PFC subregions (PrL, IL, OFC) IBA-1 fluorescence (A-C) and % hypertrophied microglia (D-F), and adiposity (white adipose tissue mass – WAT) in mice exposed to a HFHS diet across adolescence or as adults. R^2 values presented, *p<0.05, **p<0.01, ***p<0.001.

Supplementary figures

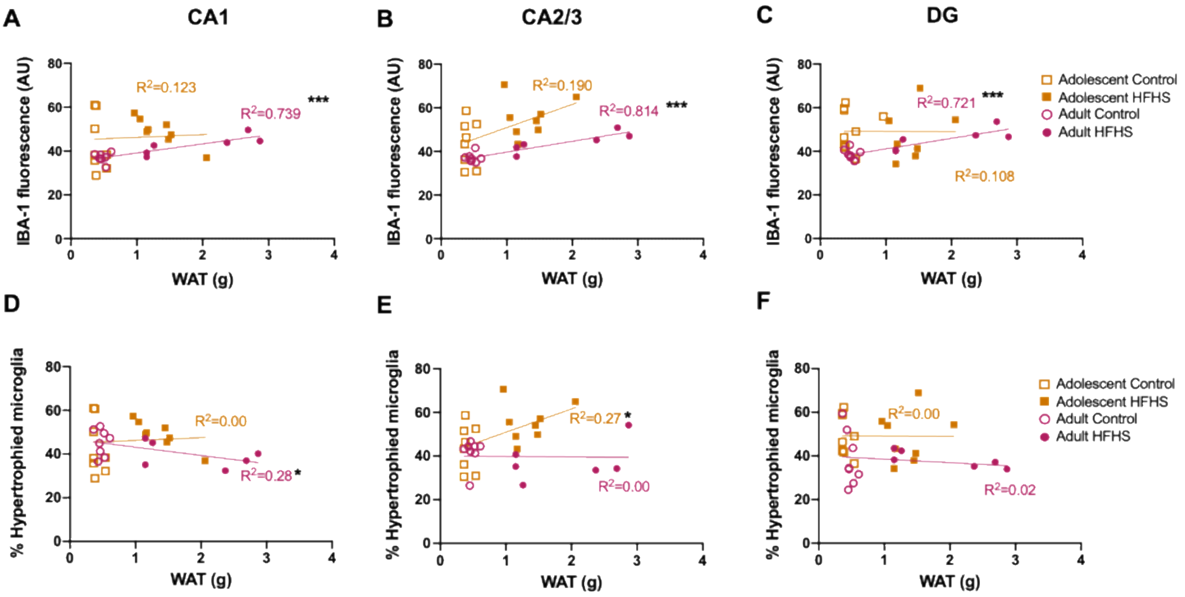

Figure S4 - Linear regressions showing the association between hippocampal subregions (CA1, CA2/3, DG) IBA-1 fluorescence (A-C) and % hypertrophied microglia (D-F), and adiposity (white adipose tissue mass – WAT) in mice exposed to a HFHS diet across adolescence or as adults. R^2 values presented, *p<0.05, **p<0.01, ***p<0.001.

Supplementary figures

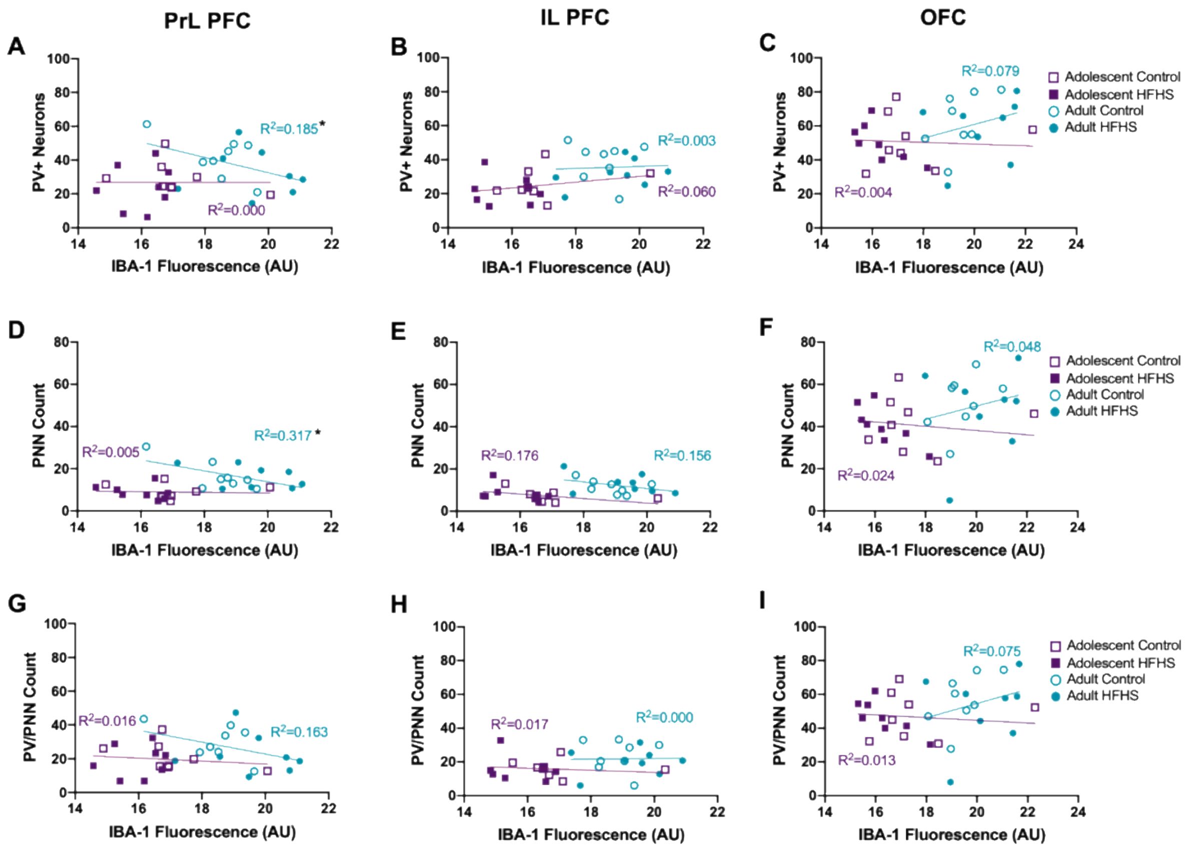

Figure S5 - Linear regressions showing the association between PFC subregions (PrL, IL, OFC) PV+ neurons (A-C), WFA+ PNNs (D-F), PV/PNN colocalisation (G-I) and IBA-1 fluorescence in mice exposed to a HFHS diet across adolescence or as adults. R^2 values presented, *p<0.05, **p<0.01, ***p<0.001.

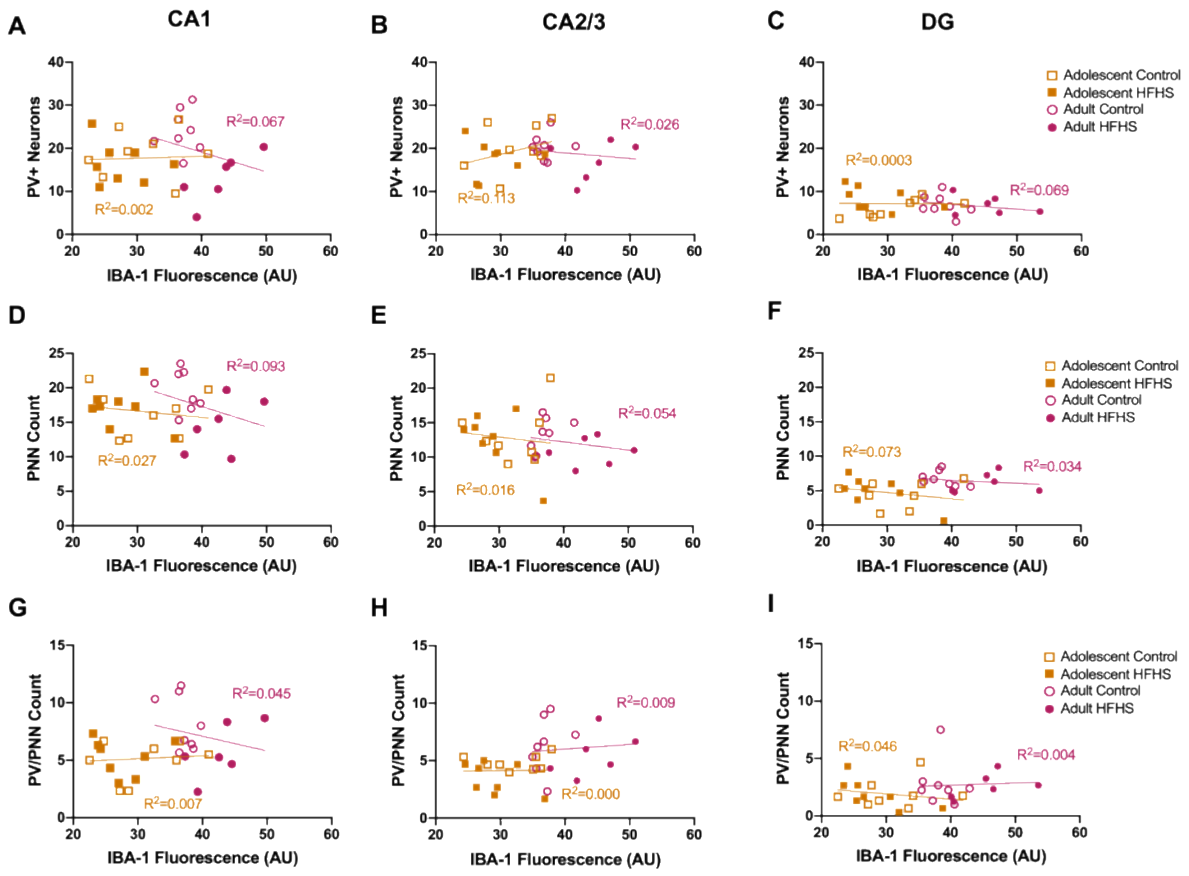

Figure S6 - Linear regressions showing the association between hippocampal subregions (CA1, CA2/3, DG) PV+ neurons (A-C), WFA+ PNNs (D-F) and PV/PNN colocalisation (G-I) and IBA-1 fluorescence in mice exposed to a HFHS diet across adolescence or as adults. R^2 values presented, *p<0.05, **p<0.01, ***p<0.001.
